# Clinical applications of genetic analysis and liquid chromatography tandem-mass spectrometry in rare types of congenital adrenal hyperplasia

**DOI:** 10.1186/s12902-021-00901-8

**Published:** 2021-11-25

**Authors:** Zhuoguang Li, Yan Liang, Caiqi Du, Xiao Yu, Ling Hou, Wei Wu, Yanqing Ying, Xiaoping Luo

**Affiliations:** 1 Department of Pediatrics, Tongji Hospital, Tongji Medical College, Huazhong University of Science and Technology, Wuhan, China; 2 Department of Endocrinology, Shenzhen Children’s Hospital, Shenzhen, China

**Keywords:** Congenital adrenal hyperplasia, Lipoid congenital adrenal hyperplasia, 11β-hydroxylase deficiency, 3β-hydroxysteroid dehydrogenase deficiency, P450 oxidoreductase deficiency

## Abstract

**Background:**

Our study aims to summarize the clinical characteristics of rare types of congenital adrenal hyperplasia (CAH) other than 21-hydroxylase deficiency (21-OHD), and to explore the clinical applications of genetic analysis and liquid chromatography tandem-mass spectrometry (LC-MS/MS) in rare CAH.

**Methods:**

We retrospectively analysed the clinical data of 5 rare cases of CAH admitted to our hospital and summarized their clinical manifestations, auxiliary examinations, diagnosis and mutational spectrum.

**Results:**

After gene sequencing, complex heterozygous variants were detected in all patients (2 cases were lipoid congenital adrenal hyperplasia (LCAH), 11β-hydroxylase deficiency (11β-OHD), 3β-hydroxysteroid dehydrogenase deficiency (3β-HSD deficiency) and P450 oxidoreductase deficiency (PORD) each accounted for 1 case), which were consistent with their clinical manifestations. Among them, 4 *novel* variants were detected, including c.650 + 2 T > A of the *StAR* gene, c.1145 T > C (p. L382P) of the *CYP11B1* gene, c.1622C > T (p. A541V) and c.1804C > T (p. Q602 *) of the *POR* gene. The LC-MS/MS results for steroid hormones in patients were also consistent with their genetic variants: 2 patients with LCAH showed a decrease in all steroid hormones; 11β-OHD patient showed a significant increase in 11-deoxycortisol and 11-deoxycorticosterone; patient with 3β-HSD deficiency showed a significant increase in DHEA; and PORD patient was mainly characterized by elevated 17OHP, progesterone and impaired synthesis of androgen levels.

**Conclusions:**

The clinical manifestations and classification of CAH are complicated, and there are cases of missed diagnosis or misdiagnosis. It’s necessary to combine the analysis of clinical manifestations and auxiliary examinations for diagnosis; if necessary, LC-MS/MS analysis of steroid hormones or gene sequencing is recommended for confirming diagnosis and typing.

**Supplementary Information:**

The online version contains supplementary material available at 10.1186/s12902-021-00901-8.

## Background

Congenital adrenal hyperplasia (CAH) is a group of autosomal recessive inherited diseases caused by congenital defects of essential enzymes in the synthesis of steroid hormones, characterized by adrenocortical insufficiency, accompanied by (or not accompanied) hyperandrogenism [1, 2]. The incidence of CAH has been reported to be approximately 1/14000–1/18000 worldwide [2] and 1/20815–1/25757 in China [3]. According to defective enzymes (genes) [4], CAH can be divided into 7 types, among which 21-OHD is the most common type (90 to 95%), 11β -OHD accounts for approximately 5–8%, 17-OHD and 3β-HSD deficiency accounts for less than 1%, and other types of CAH are relatively rare worldwide (LCAH: about 200 cases, PORD: about 130 cases, P450cc deficiency: only about 30 cases).

Due to the diversity and similarity of clinical manifestations, there are certain difficulties in the diagnosis of rare types of CAH, especially for their classification. Gene sequencing is a better verification and supplement for routine examinations, other types of CAH except 21-OHD can be well detected using next-generation sequencing technology [5, 6]. However, sometimes gene sequencing may take a long time and delay the patient’s treatment [1, 2]. Therefore, reliable clinical diagnosis is also critical. In recent years, with the application of LC-MS/MS in the detection of steroid hormones, early diagnosis and classification of CAH have become possible. In a study performed by Janzen in 2012 [7], LC-MS/MS was used to detect steroid hormone levels in patients with CAH, and they found that unlike 21-OHD patients, the 11-deoxycortisol level of patients with 11β-OHD was significantly increased, while the 21-deoxycortisol level was almost normal. It’s believe that through the analysis of the corresponding steroid hormone profile, a preliminary differential diagnosis can be made between different types of CAH.

In order to further understand the genotypic and clinical characteristics of rare types of CAH, so as to facilitate early diagnosis and treatment, 5 Chinese patients with CAH were recruited retrospectively and their clinical features were analysed.

## Methods and materials

### Subjects

According to the CAH guidelines of the Pediatric Society of Chinese Medical Association in 2016 and the American Academy of Pediatrics Endocrine in 2018(1; 2), we included 5 patients with CAH admitted to our hospital who were clinically presumed and genetically confirmed. Clinical data were collected, including history, clinical manifestations, and auxiliary examinations (adrenocortical function (including 17OHP (17α-hydroxyprogesterone), A4(androstenedione), DHEAS (dehydroepiandrosterone sulfate), T (testosterone), F (cortisol) and ACTH (adrenocorticotropic hormone), chemiluminescence immunoassay), electrolytes, hepatic and renal function, fasting glucose, etc.). Bone age assessment, adrenal Doppler ultrasound or CT and chromosome karyotype were also collected if available.

### LC-MS/MS analysis of steroid hormones

Our patients were treated with hydrocortisone (10–15 mg/m^2^/d, divided into 3–4 doses) after clinical diagnosis. During recent follow-ups, LC-MS/MS was used to detect steroid hormone levels. After obtaining informed consent, a 2 mL blood sample from each patient was sent to Kindstar Global Co., Ltd. (Wuhan) for steroid hormone detection by LC-MS/MS. First, after obtaining serum samples isolated from blood samples (3000 r/min, 3 min), 8 internal standard solutions (progesterone-d9, 17α-OH-progesterone-d8, testosterone-d3, DHEA-d6, aldosterone-d4, corticosterone-d4, cortisol-d4, deoxycortisone-d5) and MTBE (methyl tertiary butyl ether) were added to serum samples for extraction. Next, the supernatant of the above mixture was obtained by shaking, mixing and freezing centrifugation (10,000 r/min, 3 min), and then the supernatant was blown dry by the nitrogen blowing instrument. Finally, 50% methanol-aqueous solution was added to the above sample for reconstitution, the supernatant was obtained after centrifugation (10,000 r/min, 3 min) and used for subsequent chromatographic separation. The chromatographic parameters were as follows: ①Separation column: Shim-pack GIST C18 (2 μm, 100*2.1 mm); ②Mobile phase: solvent A (0.1% formic acid solution) and solvent B (0.1% formic acid-methanol solution), the gradient elution mode was used. Mass spectrometry analysis: Electrospray ionization (ESI) mode was used for ionization, positive ion multiple reaction monitoring mode (MRM) was used for scanning, and quantitative analysis was performed using the internal standard method. Finally, in the data analysis, Lab Solutions software was used to analyse the hormone levels of the patient’s serum sample.

### Genetic analysis

After obtaining informed consent, gene sequencing (Beijing MyGenostics Inc.) was performed on the probands and their parents. In addition to 7 known CAH genes (*CYP21A2*, *StAR*, *CYP11B1*, *HSD3B2*, *POR*, *CYP17A1* and *CYP11A1*), 37 adrenal or gonadal genes including *NR0B1*, *PRKACA*, *DHCR7*and so on. were also tested to exclude the possibility of adrenal or gonadal abnormalities (see Supplementary Table 1). First, we fragmented 1–3 μg of genomic DNA, which was extracted from the sample, to an average size of 180 bp with a Bioruptor sonicator (Diagenode). The preparation of DNA Library carried out by Illumina protocols mainly included end repair, adapter ligation and PCR enrichment. The amplified DNA was captured using a GenCap CAH capture kit (MyGenostics GenCap Enrichment Technologies). The DNA probes were designed to tile along the exon regions and exon–intron boundaries of the 44 target genes. After enrichment of DNA fragments, an Illumina HiSeq X sequencer was used for high-throughput sequencing of the captured exon region. Suspected candidate variants were screened by comprehensively considering the genetic pattern of the disease and the patient’s clinical characteristics. The pathogenicity of variants was predicted according to the 2015-ACMG Standards and Guidelines [8].

## Results

After gene sequencing, of the 5 patients with CAH, 2 cases were LCAH, 11β-OHD, 3β-HSD deficiency and PORD each accounting for 1 case. See Table 1.

**Table 1:** Clinical characteristics of 5 patients with rare types of CAH

Types	Genes	Variants	Inheritance	Pathogenicity	First visit (years)	Complaint	Adrenocortical function*
LCAH	*StAR*	(1) c.650 + 2 T > A #, c.814C > T (p. R272C); (2)c.707_708delAGinsCTT (p.K236Tfs), c.772C > T (p.Q258*)	(1) Paternal, Maternal; (2) Paternal, Maternal	(1) LP, LP; (2) P, LP	Neonatal	cyanosis and skin pigmentation throughout the body	17OHP ↓/N, A4 ↓, DHEAS N, F ↓/N, ACTH ↑↑
11β-OHD	*CYP11B1*	c.422G > A(p.R141*), c.1145 T > C(p.L382P) #	Maternal, Paternal	LP, VUS	3.5	enlarged penis for 3 years	17OHP ↑, A4 ↑↑, DHEAS ↑, T ↑, F N, ACTH ↑
3β-HSD deficiency	*HSD3B2*	c.674 T > A(p.V225D), c.776C > T(p.T259M),	Maternal, Paternal	P, P	9	enlarged testicles for 5 years	17OHP ↑, A4 ↑↑, DHEAS ↑↑, T ↑, F ↓, ACTH ↑↑
PORD	*POR*	c.1622C > T(p.A541V) #, c.1804C > T(p.Q602*) #	Maternal, Paternal	LP, LP	2	external genital abnormalities for more than 1 year	17OHP ↑, A4 ↓↓, T ↓, F N, ACTH ↓

Note: *See Supplementary Table 2 for details. # novel variants haven’t been reported before; *P* pathogenic; *LP* likely pathogenic; *VUS* variant of uncertain significance; *17OHP* 17α-hydroxyprogesterone; *A4* androstenedione; *T* testosterone; *DHEAS* dehydroepiandrosterone sulfate; *ACTH* adrenocorticotropic hormone; *F* cortisol; ↑, increase; ↓, decrease; ↑↑ or ↓↓, beyond the limits of detection; N, normal

### Clinical manifestations

The 2 patients with LCAH were admitted in the neonatal period due to “cyanosis and skin pigmentation throughout the body”. The male patient also had occult penis and hypospadias, while no abnormalities in the vulva were found in the female patient.

The 11β-OHD patient was first admitted at 3.5 years old due to “enlarged penis for 3 years”. He had obvious acne on his face and chest, and obvious pigmentation on his vulva, accompanied by accelerated growth of height (114.4 cm, + 3.54 SDS). Blood pressure monitoring showed: 107–111/55–79 mmHg. His twin brother also had an enlarged penis at birth and died prematurely of an infection.

The patient with 3β-HSD deficiency was admitted at 9 years old due to “enlarged testicles for 5 years”(>20 mL). He had obviously swarthy skin throughout the body, accompanied by accelerated growth of height (143.3 cm, + 1.36 SDS). His elder brother was also diagnosed with 3β-HSD deficiency, with enlarged testicles and swarthy skin, and his final adult height was 150.3 cm (− 3.71 SDS).

The PORD patient was admitted at 2 years old due to “external genital abnormalities for more than 1 year”. An enlarged clitoris like a penis was found, and one shared opening for her vagina and urethra, but no obvious pigmentation was seen.

### Auxiliary examinations

In terms of adrenocortical function (chemiluminescence immunoassay), LCAH patients manifested as a low level of all adrenal hormones with a significant increase in ACTH; 11β-OHD patient was mainly characterized by elevated 17OHP and androgen; 3β-HSD deficiency patient was characterized by a significant increase in DHEAS; and patient with PORD suffered 17OHP increasing slightly with normal androgen levels. See Table 1 and Supplementary Table 2 for details. The main biochemical indices of these 5 patients, such as electrolytes (Na+, K+), hepatic and renal function and son on, were basically normal at the first visit.

In terms of BA assessment, BA of 11β-OHD (Chronological Age/CA: 3.5 years, BA: 13 years) and 3β-HSD deficiency (CA: 9 years, BA: 16 years) patients significantly advanced, resulting in their HtSDS_BA_ fell far behind (− 5.72 SDS for 11β-OHD, − 4.56 SDS for 3β-HSD). However, the BA of PORD patient and 1 case of LCAH lagged behind their CA by 1 year, and another LCAH patient didn’t undergo BA assessment because of her young age (< 2 years).

#### LC-MS/MS analysis of steroid hormones

**Fig. 1:**
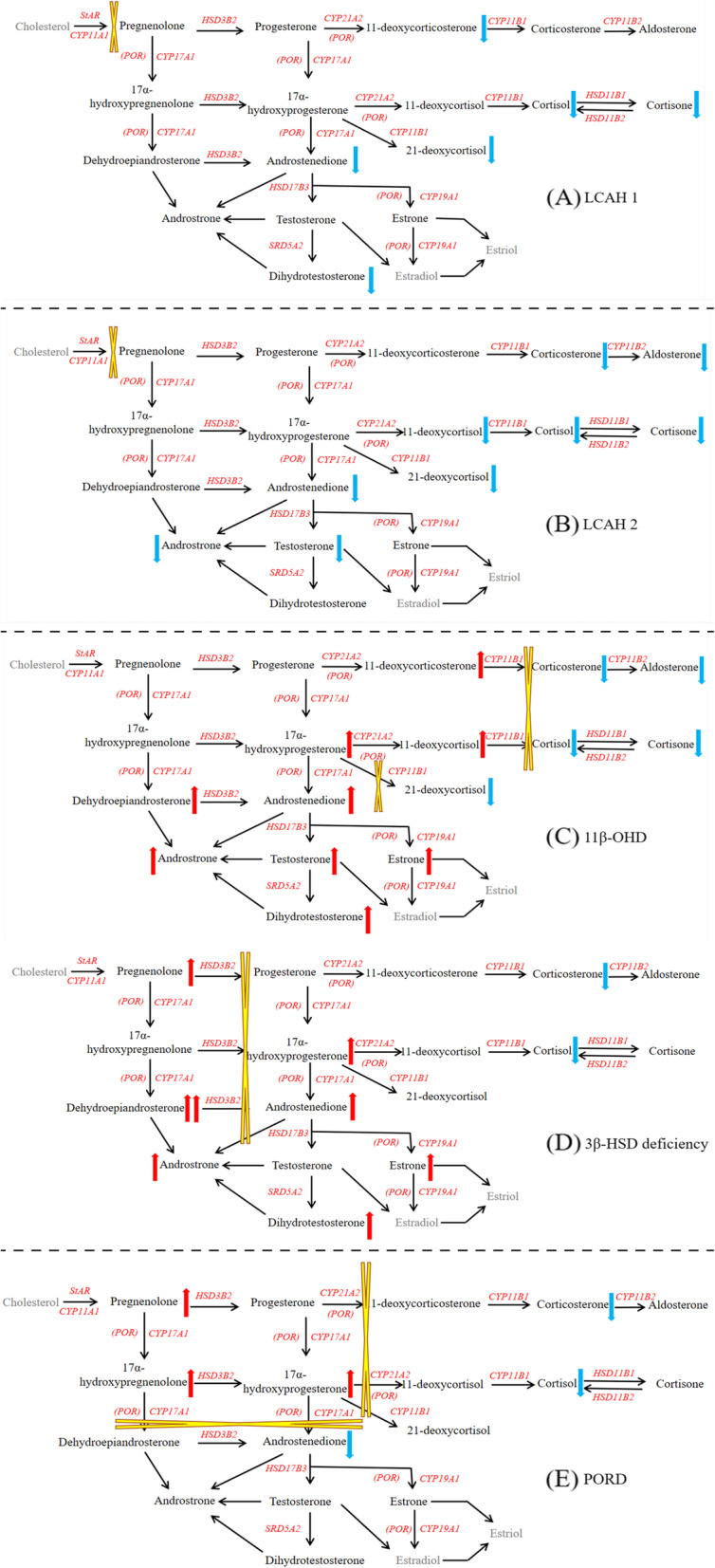
LC-MS/MS analysis of steroid hormones for 5 patients with rare types of CAH (1A. LCAH 1; 1B. LCAH 2; 1C. 11β-OHD; 1D. 3β-HSD deficiency; 1E. PORD)

During recent follow-ups, LC-MS/MS was used to detect steroid hormone levels. See Supplementary Table 3 and Fig. 1 for details.

The 2 patients with LCAH showed a decrease in the levels of all steroid hormones because of the defect of steroidogenic acute regulatory protein (StAR protein). Because of 11β-hydroxylase deficiency, 11β-OHD patient showed characteristic changes: 11-deoxycortisol and 11-deoxycorticosterone levels were significantly increased, accompanied by increasing levels of progesterone, androgen and estrogen, while the levels of 21-deoxycortisol, glucocorticoids and mineralocorticoids decreased. In patient with 3β-HSD deficiency, DHEA was significantly increased, accompanied by increased 17OHP, progesterone and androgen, while the levels of glucocorticoids and mineralocorticoids were decreased. Patient with PORD was characterized by increased 17OHP and progesterone, while the levels of androgen and glucocorticoids were decreased.

### Genetic characteristics

After gene sequencing, complex heterozygous variants were detected in all patients (2 cases were LCAH, 11β-OHD, 3β-HSD deficiency and PORD each accountingfor 1 case). Among them, one *novel* variant was detected in the *StAR* gene (c.650 + 2 T > A); one *novel* variant c.1145 T > C (p. L382P) was detected in the *CYP11B1* gene; and two *novel* variants were detected in the *POR* gene, that is c.1622C > T (p. A541V) and c.1804C > T (p. Q602 *). According to the 2015-ACMG Standards and Guidelines, most of the variants in these patients were pathogenic or likely pathogenic variants, which were consistent with their clinical manifestations. See Table 1.

## Discussion

Congenital adrenal hyperplasia (CAH) is a group of diseases caused by steroid hormone synthesis disorders. From 1955 to 2004, the genes for most of the essential enzymes in the steroid hormone synthesis pathway were successively cloned. According to the types of known defective enzymes (genes), CAH can be divided into 7 types [9–14], including 21-OHD (encoded by *CYP21A2* gene), 11β-OHD (encoded by *CYP11B1* gene), 17-OHD (encoded by *CYP17A1* gene), 3β-HSD deficiency (encoded by HSD3B2 gene), LCAH (encoded by *StAR* Gene), PORD (encoded by *POR* gene) and cholesterol side-chain cleavage enzyme deficiency (P450cc deficiency, encoded by *CYP11A1* gene).

In steroid hormone cells, adrenal steroid hormone production is a dynamic process that relies on the de novo synthesis of cholesterol in response to the stimulation of ACTH and other regulatory factors [14, 15]. The first step in steroid production is also the rate-limiting step, cholesterol is first converted to pregnenolone (Preg) by the cholesterol side-chain cleavage enzyme (*CYP11A1*). This process also relies on the transmembrane transport of cholesterol from the outer mitochondrial membrane to the inner membrane by steroidogenic acute regulatory protein (*StAR*). Then, the 17α-hydroxylase activity of P450C17 (*CYP17A1*) converts Preg to 17α-hydroxypregnenolone (17OHP5). Preg and 17OHP5 are synthesized by 3β-hydroxysteroid dehydrogenase 2 (*HSD3B2*) to produce progesterone and 17OHP, respectively. The latter is catalysedby a series of enzymes such as 21-hydroxylase (*CYP21A2*) and 11β-hydroxylase (*CYP11B1*), and then finally produces mineralocorticoids and glucocorticoids.

CAH caused by 11β-OHD and 3β-HSD deficiency is characterized by hyperandrogenemia, and the clinical manifestations are similar to those of 21-OHD. However, PORD and LCAH may lead to abnormal sexual development and a lack of secondary sexual characteristics due to androgen synthesis disorders. In our study, 2 cases of LCAH were admitted in the neonatal period for “lip cyanosis and skin pigmentation”, and were initially misdiagnosed as cardiovascular disease or Addison syndrome initially. Patients with 11β-OHD and 3β-HSD deficiency were diagnosed with CAH with hyperandrogenism symptoms, and both of them suffered from an enlarged penis, rapid height growth and backward HtSDS_BA_; it was not until genetic tests were carried out that their specific disease type was identified. For patient with PORD, she was admitted with “external genital abnormalities” and initially diagnosed with “atypical 21-OHD” initially. Recently, after careful study of her medical history, we felt it’s necessary to perform gene sequencing (long PCR + NGS + MLPA) again, and finally confirmed the pathogenic variants of the *POR* gene. Pathogenic or possible pathogenic variants were detected in all patients, which were consistent with their clinical manifestations. Among them, one *novel* variant was detected in the *StAR* gene (c.650 + 2 T > A); one *novel* variant c.1145 T > C (p. L382P) was detected in the *CYP11B1* gene; and two *novel* variants were detected in the *POR* gene, that is c.1622C > T (p. A541V) and c.1804C > T (p. Q602 *).

The concentration of steroid hormones in organisms is very low, generally calculated in units of nmol/L and pmol/L, and there are many kinds of steroid compounds with similar structure [16–18]. Due to the influence of antigen-antibody cross reaction and poor specificity, the detection result of steroid hormones by immunoassay might be higher than the actual level, which may lead to improper drug replacement therapy. In many complex adrenal diseases, including CAH, the ratio or relative change of multiple steroid hormones is more important than the value of any one steroid hormone [19, 20]. In recent years, with the widespread application of LC-MS/MS in clinical medicine [21–23], LC-MS/MS can detect nearly 20 types in steroid hormones at the same time. Through the relative changes of hormone levels, it can initially verify the diagnosis and classification of CAH, which is helpful for early diagnosis and timely treatment [24, 25]. In our study, the LC-MS/MS results for steroid hormones in patients were also consistent with their genetic variants: 2 patients with LCAH showed a decrease in all steroid hormones; 11β-OHD patient showed a significant increase in 11-deoxycortisol and 11-deoxycorticosterone; patient with 3β-HSD deficiency showed a significant increase in DHEA; and PORD patient was mainly characterized by elevated 17OHP, progesterone and impaired synthesis of androgen levels. The above results indicate that the results of LC-MS/MS in patients with CAH are in good agreement with the results of gene sequencing; through the change spectrum of the steroid hormones, we can preliminarily judge the possible positions of defective enzymes in CAH patients and make an initiatory judgement on its typing.

Due to the location of enzyme defects, the clinical manifestations of different types of CAH are complex and changeable, the prognosis and treatments will also be different. CAH caused by 11β-OHD and 3β-HSD is often characterized by hyperandrogenism, which is likely to cause problems such as advanced BA and precocious puberty and may need to be treated with gonadotrophin-releasing hormone agonists [26]; while PORD and LCAH often have androgen synthesis disorders and then lack of secondary sexual characteristics, they may require sex hormone to induce development after puberty. Therefore, the clinical diagnosis of CAH cannot be too indistinct, and it’s necessary to clarify the specific classification to better judge the prognosis and guide treatment.

## Conclusions

Our study summarized the clinical characteristics (clinical manifestations, auxiliary examinations and diagnosis, etc.) of 5 cases of rare CAH. In our study, we detected 4 *novel* variants that have not been reported before, which enriched the variant spectrum of the CAH gene. We also found that compared with immunoassay, the use of LC-MS/MS to detect steroid hormones can greatly improve the positive predictive value of CAH diagnosis, and the comprehensive analysis of multiple steroid hormones by LC-MS/MS can also help in the diagnosis and classification of CAH. The treatment goal of CAH has gradually changed from preventing the occurrence of crisis to ensuring normal growth and development as much as possible. It’s believed that LC-MS/MS will play an increasingly important role in the clinical application of CAH with further research.

## Supplementary Information

**Additional file 1:.**

**Additional file 2:.**

**Additional file 3:.**

## Data Availability

The dataset analyzed in the current study is available from the corresponding author upon reasonable request.

## Abbreviations

CAH: Congenital adrenal hyperplasia
21-OHD: 21-hydroxylase deficiency
11β-OHD: 11β-hydroxylase deficiency
3β-HSD deficiency: 3β-hydroxysteroid dehydrogenase deficiency
LCAH: Lipoid congenital adrenal hyperplasia
PORD: P450 oxidoreductase deficiency
StAR: Steroidogenic acute regulatory protein
BA: Bone age
HtSDS_BA_: Height standard deviation score for BA
PCR: Polymerase chain reaction
NGS: Next-generation sequencing
ACTH: Adrenocorticotropic hormone
F: Cortisol
17OHP: 17α-hydroxyprogesterone
A4: Androstenedione
T: Testosterone
DHEAS: Dehydroepiandrosterone sulfate
