# Supplementary material for: Clinical applications of genetic analysis and liquid chromatography tandem-mass spectrometry in rare types of congenital adrenal hyperplasia
Source: BMC Endocr Disord. 2021 Nov 25;21:237. doi: 10.1186/s12902-021-00901-8 (PMC8620188; doi:10.1186/s12902-021-00901-8)
Supplement: Supplementary file 1 — Additional file 1:. [file 12902_2021_901_MOESM1_ESM.docx]

**Supplementary Table 1.** Analyzed genes of congenital adrenal hyperplasia panel

| Items | Analyzed genes |
| --- | --- |
| CAH genes | *CYP21A2、StAR、CYP11B1、HSD3B2、POR、CYP17A1、CYP11A1* |
| Adrenal/gonadal genes to identify CAH | *NR0B1、PRKACA、DHCR7、GK2、PDE8B、LHX4、ARMC5、MC2R、GK、H6PD、CDKN1C、ABCD1、SOX3、GNAS、MRAP、POMC、HSD11B1 MKS1、NNT、TBX19、MEN1、MCM4、REN、NR5A1、AIRE、NR3C1、PCSK1、TXNRD2、RXRA、PRKAR1A、HESX1、GLCCI1、TP53、CYP11B2、RXRB、PDE11A、PROP1* |
