# Supplementary material for: Clinical applications of genetic analysis and liquid chromatography tandem-mass spectrometry in rare types of congenital adrenal hyperplasia
Source: BMC Endocr Disord. 2021 Nov 25;21:237. doi: 10.1186/s12902-021-00901-8 (PMC8620188; doi:10.1186/s12902-021-00901-8)
Supplement: Supplementary file 2 — Additional file 2:. [file 12902_2021_901_MOESM2_ESM.docx]

**Supplementary Table 2.** Adrenocortical function of 5 patients with rare types of CAH (Chemiluminescence Immunoassay)

|  | 17OHP  (nmol/L) | A4  (nmol/L) | DHEAS  (nmol/L) | T  (nmol/L) | F (nmol/L) | ACTH (pg/ml) |
| --- | --- | --- | --- | --- | --- | --- |
| LCAH 1 | 0.45↓ | 0.06↓ | 50.20 | - | 68.40↓ | ≥1250.00 |
| LCAH 2 | 1.82 | 2.75 | 53.32 | - | 171.98 | ≥1250.00 |
| 11β-OHD | 58.10↑ | ≥35.00 | 1090.00↑ | 13.72↑ | 137.83 | 427.00↑ |
| 3β-HSD deficiency | 36.36↑ | ≥35.00 | ≥21700 | 7.20↑ | 128.80↓ | ≥1250.00 |
| PORD | 12.73↑ | ＜0.01 | - | 0.02↓ | 325.00 | 18.60↓ |

Note: Hormone levels should be read according to the recommended reference range for laboratory use.
