# Supplementary material for: Clinical applications of genetic analysis and liquid chromatography tandem-mass spectrometry in rare types of congenital adrenal hyperplasia
Source: BMC Endocr Disord. 2021 Nov 25;21:237. doi: 10.1186/s12902-021-00901-8 (PMC8620188; doi:10.1186/s12902-021-00901-8)
Supplement: Supplementary file 3 — Additional file 3:. [file 12902_2021_901_MOESM3_ESM.docx]

**Supplementary Table 3.** LC-MS/MS analysis of steroid hormones for 5 patients with rare types of CAH

| steroid hormones (ng/ml) | LCAH 1 | LCAH 2 | 11β-OHD | 3β-HSD deficiency | PORD |
| --- | --- | --- | --- | --- | --- |
| Sex | Male | Female | Male | Male | Female |
| Age(years) | 10 | 1.6 | 4.08 | 12 | 10.92 |
| Pregnenolone | 0.157 | 0.237 | 0.474 | 2.639↑ | 0.925↑ |
| Progesterone | 0.112 | 0.357 | 0.386 | 0.33 | 2.511 |
| 11-deoxycorticosterone | ＜0.02↓ | 0.023 | 5.431↑ | 0.053 | 0.066 |
| Corticosterone | 0.036 | 0.122↓ | 19.135 | 0.249↓ | 7.290 |
| Aldosterone | 0.064 | 0.009↓ | 0.018↓ | 0.097 | 0.090 |
| 17α-hydroxypregnenolone | 0.681 | 0.595 | 0.475 | 0.506 | 2.618↑ |
| 17α-hydroxyprogesterone | 0.263 | 0.111 | 5.597↑ | 5.716↑ | 4.985↑ |
| 11-deoxycortisol | 0.155 | 0.054↓ | 93.264↑ | 1.848 | 0.956 |
| 21-deoxycortisol | ＜0.125↓ | 0.067↓ | 0.129↓ | 0.337 | 0.409 |
| Cortisol | 5.081↓ | 1.843↓ | 3.427↓ | 16.122↓ | 16.930↓ |
| Cortisone | 3.045↓ | 0.739↓ | 1.780↓ | 6.943 | 4.904↓ |
| Dehydroepiandrosterone | 0.378 | 0.114 | 0.891↑ | 50.826↑ | 0.289 |
| Androstenedione | 0.058↓ | 0.044↓ | 4.104↑ | 2.936↑ | 0.065↓ |
| Testosterone | 0.055 | 0.004↓ | 1.173↑ | 1.976 | 0.091 |
| Dihydrotestosterone | ＜0.05↓ | 0.052 | 1.256↑ | 2.135↑ | 0.082 |
| Androstrone | 0.206 | 0.123↓ | 0.850↑ | 1.224↑ | 0.537 |
| Estrone | 0.038 | 0.029 | 0.074↑ | 0.033↑ | 0.071 |
